# Prevalence, antimicrobial susceptibility patterns and associated risk factors of *Shigella* and *Salmonella* among food handlers in Arba Minch University, South Ethiopia

**DOI:** 10.1186/s12879-016-2035-8

**Published:** 2016-11-21

**Authors:** Mohammedaman Mama, Getaneh Alemu

**Affiliations:** Department of Medical Laboratory Sciences, College of Medicine and Health Sciences, Arba Minch University, Arba Minch, Ethiopia

**Keywords:** Food handlers, Risk factors, *Salmonella*, *Shigella*

## Abstract

**Background:**

The availability of safe food improves health of the people that contributes to productivity and provides an effective platform for development and poverty alleviation. On the other hand, unsafe food handling and processing can serve as a vehicle for the transmission of a variety of disease causing agents. The risk of food getting contaminated depends largely on the health status of the food handlers, their personal hygiene, knowledge and practice of food hygiene. Food borne diseases are therefore a public health problem in developed and developing countries which is also true for Ethiopia. Hence, the aim of this study was to determine prevalence, antimicrobial susceptibility patterns and associated risk factors of *Shigella* and *Salmonella* among food handlers in Arba Minch University, South Ethiopia.

**Methods:**

A cross sectional study was conducted among food handlers in Arba Minch University students’ cafeteria from April- June, 2015. Structured questionnaire was used to collect socio demographic data and associated risk factors. Stool sample was collected and examined for pathogens following standard procedures. Biochemical tests were done to identify the species of pathogens and sensitivity test was done using Kirby- Baur disk diffusion technique.

**Results:**

A total of 376 food handlers were enrolled in the study with the response rate of 100% for data collected by questionnaire. About 7.4% were aged less than 20 years with majority (63.3%) lay in the working age group of 21-35 years. However, a total of 345 food handlers participated for stool examination of whom, stool cultures revealed 6.9% of *Salmonella* and 3% *Shigella* isolates. Finger nail status (AOR=0.033), hand washing practice after toilet (AOR= 0.006) and touching food with bare hands (AOR= *p* < 0.001) were independent predictors of infectious enteric diseases among the food handlers. All isolated pathogens were resistant to amoxicillin (100%), followed by clarithromycin (41%) and amoxicillin-clavulanic acid (35%).

**Conclusion:**

The present study showed high prevalence of enteropathogens among the study participants. The study also revealed poor personal hygiene like poor practice of hand washing. Therefore, pre placement, in service training on personal and food hygiene should be provided to all food handlers with regular sanitary inspection to improve adherence of food handlers to personal hygiene and food safety practices.

## Background

Food borne diseases are a public health problem in developed and developing countries. The World Health Organization (WHO) estimated that in developed countries, up to 30% of the population suffer from food borne diseases each year, whereas in developing countries up to 2 million deaths are estimated per year [1, 2]. The problem is more severe in developing countries like Ethiopia where there is lack of personal hygiene, food safety measures and reliable statistics on food borne diseases are not available due to poor or non-existent reporting systems [3, 4].

Food-borne diseases are a serious threat to people in Africa, causing an unbearable public health burden and massive economic losses. According to WHO most recent estimates, 700,000 deaths per year in Africa are due to food and water-borne related diseases. These outbreaks only show the tip of the iceberg, as many more cases that are sporadic went unreported [5].

Numerous studies have been conducted to assess enteropathogens infection among food handlers in Africa including different regions of Ethiopia. In Ethiopia, the rate of infection with *Salmonella* and *Shigella* among food handlers ranged from 1 to 7.5% [6–9].

Food borne Salmonellosis often follows consumption of contaminated animal products such as raw meat, poultry and eggs. Not washing fresh fruits and vegetables before eating them, as well as not thoroughly cleaning work surfaces used to prepare raw meat and other foods in the kitchen can also be source of *Salmonella* [4, 10].

In Ethiopia, minced beef is usually used for the preparation of a popular traditional Ethiopian dish known as locally “KITFO” and most of the time it is consumed medium cooked. The habit of raw meat consumption and the presence of *Salmonella* in minced beef indicates, in addition to the poor hygienic standards in food handling in the country, the presence of great public health hazards of *Salmonella* [11, 12]. Moreover, it is well known habit to eat raw food in the study area known as locally “KURTE” which doubles public health hazards of *Salmonella* in the study area. According to report [13], in recent years the number of out breaks of *Salmonella* in humans and its antimicrobial resistance has increased considerably in the country [14]. Despite the challenge, research conducted about food Salmonellosis among food handlers and its antimicrobial susceptibility pattern is scarce in Ethiopia particularly in Arba Minch.

Shigellosis is a significant problem around the world with the highest prevalence in tropical and subtropical parts of the world where living standards are very low and access to safe and adequate drinking water and proper excreta disposal systems are often limited [15]. This is an indication for Shigellosis to be public health problem in Ethiopia, where there is substandard hygiene and unsafe water supplies [16, 17]. Another alarming egression for these bacteria is the emergence of antimicrobial resistant species that was indicated in a few studies conducted in the country [18, 19]. Since the prevalence and pattern of resistance of *Shigella* species in the country varies from one area to another, updated information on their prevalence and resistance patterns is very important for the proper selection and use of antimicrobial agents in a setting. Thus, this study was aimed to determine prevalence, antimicrobial susceptibility patterns and associated risk factors of *Shigella* and *Salmonella* among food handlers in Arba Minch University, South Ethiopia.

## Methods

### Study area, design and period

A cross sectional study design was conducted at Arba Minch University, which is located 505 km south of Addis Ababa in the vicinity of unique natural and anthropogenic diversity, from April to June, 2015.

### Sample size determination and sampling technique

The sample size was determined using sample size determination for estimation of single population proportion formula. Taking 0.63 proportion value, 95% confidence interval (z = 1.96) and 5% marginal error (d = 0.05) the initial sample size was 358 and, finally by considering a 5% (≈18 subjects) non response rate, the final sample size was determined to be 376. To select representative groups a proportional sample size was determined for each stratum, and food handlers were selected randomly by lottery method from the roster lists of food handlers which was obtained from cafeteria office of each Arba Minch University campuses.

### Data collection and Laboratory processing

Data related to socio-demographic characteristics, and personal hygiene practices of food handlers and related risk factors were collected by face to face interview using pre tested structured questionnaire. All the questionnaires were checked for accuracy and completeness. After proper instruction, food handlers were given labeled collection cups that contain applicator sticks. From each, about 2 g of fresh stool was collected. Each of the specimens was checked for its label, quantity and procedure of collection and transported with Cary-Blair transport media (Oxoid).

### Culture and identification

The collected samples were immediately processed for bacteriological analysis. Stool swabs were inoculated in to Selenite F broth (Oxoid) and incubated for 24 h at 37 °C followed by sub culturing on Xylose lysine deoxycholate agar plates (Oxoid) and re incubated for 24 h at 37 °C. Examination of the plates for significant colonies of *Salmonella* and *Shigella* species was done. An inoculum of each stool specimen was made on Blood agar and MacConkey agar plates (Oxoid) using cotton swab and then streaked with sterile inoculation loop. The plates were incubated at 37 °C for 24–48 h. Preliminary identification of pathogens was based on colony characteristics of the organisms and motility tests. Biochemical tests (Oxoid) were also carried out by inoculation of Kliger iron agar, Lysine iron agar, simmons citrate agar and media for indole and urease production for final identification.

### Antimicrobial susceptibility test

Antimicrobial susceptibility test was performed by Kirby-Bauer disk diffusion technique according to criteria set by Clinical Laboratory Standard Institute (CLSI), 2012. The inoculum was prepared by picking parts of similar test organisms with a sterile wire loop suspended in sterile normal saline. The density of suspension to be inoculated was determined by comparison with opacity standard on McFarland 0.5 Barium sulphate solution. The test organisms were uniformly seeded over the Mueller-Hinton agar (Oxoid) surface and exposed to a concentration gradient of antibiotic diffusing from antibiotic-impregnated paper disk into the agar medium, and then incubated at 37 °C for 24 to 48 h. Diameters of the zone of inhibition around the discs was measured to the nearest millimeter using a ruler and classified as sensitive, intermediate, and resistant according to the standardized table supplied by CLSI, 2012. The antibiotic disks tested were amoxicillin (10 μg), gentamicin (10 μg), chloramphenicol (30 μg), kanamycin (30 μg), trimethoprim sulfamethoxazole (25 μg), amoxicillin-clavulanic acid (30 μg), clarithromycin (30 μg) and ceftriaxone (30 μg).

### Data quality

Data quality was ensured at various activities of the study by following prepared standard operating procedure (SOP). One week prior to the actual data collection period, pre-test was conducted on 5% food handlers working in cafeteria of Arba Minch hospital. The questionnaire was translated to local language and back translated to English to ensure the consistency of the questionnaires. Completed questionnaires were checked and corrected on daily base. Well experienced and trained laboratory technologist was recruited for laboratory examination. Culture Media was prepared according to manufacturer’s instruction and sterility was checked by incubating representative of the batch at 35–37 °C overnight and observing pathogens growth. Those batches of the media that showed growth were discarded. Control strains were used for aerobic *P. aeruginosa* (ATCC-27853), for Gram positives *S. aureus* (ATCC-25923) and for Gram negatives *E. coli* (ATCC-25922) as a quality control for culture and potency of antibiotic disks.

### Data analysis

Data was edited, cleaned, entered and analyzed using statistical package for social science (SPSS) version 20. Descriptive analysis such as frequencies and mean was used. Initially the association between each exposure and the presence of infection was assessed using the chi-square test, and odds ratio was computed to measure the strength of the association. To determine independent risk factors for those having association during chi-square test, logistic regression analysis was employed. *P*-value of ≤ 0.05 was considered to indicate statistically significant association. Finally the result was presented using tables.

## Results

### Socio demographic characteristics

In the present study, majority of the infected food handlers were females. The highest proportion of infection (64.7%) was seen among age group of 21–35 years for both pathogens. Of the total isolated pathogens, 5.8% were illiterate, while 35.3% was associated with primary school as shown in Table 1. The isolation rate of both pathogens was higher among food handlers who served for a period of 1–5 and 6–10 years (32.4%). All of the food handlers were not trained about food handling, preparation and none of them had periodic medical check-up.

**Table 1:** The prevalence of pathogens infection (*N* = 34) with respect to socio-demographic characteristics of food handlers at Arba Minch University students’ cafeteria, Arba Minch, South Ethiopia, April- June 2015

Demographic characters	Total Infected No. (%)	*Salmonella* positive No. (%)	*Shigella* positive No. (%)
Sex			
Male	12 (35.3)	8 (66.7)	4 (33.3)
Female	22 (64.7)	16 (72.8)	6 (27.2)
Age in years			
≤ 20	2 (5.9)	2 (100)	0
21–35	22 (64.7)	14 (63.6)	8 (36.4)
36–50	10 (29.4)	8 (80)	2 (20)
Years of service			
< 1 year	3 (8.7)	3 (100)	0
1–5 years	11 (32.4)	9 (81.8)	2 (18.2)
6–10 years	11 (32.4)	6 (54.5)	5 (45.5)
> 10 years	9 (26.5)	6 (66.7)	3 (33.3)
Educational status			
Illiterate	2 (5.8)	2 (100)	0
Primary school	12 (35.3)	10 (83.3)	2 (16.7)
Secondary school	9 (26.5)	6 (66.7)	3 (33.3)
Higher than secondary school	11 (32.4)	6 (54.5)	5 (45.5)
Certified in food training			
No	34 (100)	24 (71)	10 (29)
Yes	0	0	0

### Hand washing practices

As it can be seen from Table 2, there are self-reported practices of hand washing with soap and water on different occasions. About 17.3% of food handlers never washed their hands after touching body parts and 13.3% never washed hands after blowing nose. A large number of food handlers reported that they wash their hands only by water and all of them wash their hands either with water alone or with soap after toilet and touching dirty materials.

**Table 2:** Hand washing practices of food handlers (*N* = 376) at Arba Minch University Students’ Cafeteria, Arba Minch, South Ethiopia, April–June 2015

Hand washing practices	No (%)	Only with water (%)	With water and soap (%)
After going to toilet	0	164 (43.6)	212 (56.4)
Before food handling	4 (1.1)	199 (52.9)	173 (46)
In-between handling raw & cooked food	14 (3.7)	270 (71.8)	92 (24.5)
After blowing nose	50 (13.3)	298 (79.3)	28 (7.4)
After touching body parts	65 (17.3)	253 (67.3)	58 (15.4)
After touching dirty materials	0	261 (69.4)	115 (30.6)

### Pathogens prevalence and associated risk factors

A total of 345 food handlers participated for stool examination. Of this *Salmonella* species accounted 6.9% (*24/345)* and *Shigella* species accounted 3% (10/345), while no pathogens was isolated from the stool of 311 (90.1%) participants. Different factors were assessed for possible association with pathogens infection among the study participants. Of those checked risk factors: finger nail status, hand washing practice after toilet, and transferring food with bare hands were significantly associated with infection. The logistic analysis result revealed that, pathogens infections (*p* = 0.033) were 1.84 times more likely to occur (AOR: 1.842, 95% CI [1.049–3.233]) among food handlers who had untrimmed finger nail as compared to those who trimmed (Table 3). The multivariate logistic regression model estimated that food handlers who use water only to wash their hands after toilet were 2 times (AOR: 2.08, 95% CI: 1.24–3.49) more likely to be infected with pathogens than those who use water and soap together. About 53.5% of food handlers who had touched food with bare hands were found to be infected with at least one bacterium. The practice of touching food with bare hands had a statistically significant association with pathogens infection (*p* < 0.001) (Table 3). The logistic regression model estimated that individuals who touch food with bare hands were five times (AOR: 5.34, 95% CI: 2.705–10.52) more likely to be infected with pathogens than those who do not touch food with bare hands.

**Table 3:** Multivariate logistic regression analysis: Predictors for Pathogens infection among food handler’s (*N* = 376) working in Arba Minch University Students’ Cafeteria, Arba Minch, South Ethiopia, April–June 2015

Variables	Negative No. (%)	Positive No. (%)	Adjusted OR (95% CI)	*P*-value
Finger nail status				
Not trimmed	62 (69)	28 (31)	1.842 (1.049–3.233)	0.033
Trimmed	230 (80.4)	56 (19.6)	1.00	
Hand washing after toilet				
With water only	115 (70)	49 (30)	2.079 (1.24–3.486)	0.006
With water and soap	177 (83.5)	35 (16.5)	1.00	
Touching food with bare hands				
No	20 (46.5)	23 (53.5)	1.00	0.000
Yes	272 (82)	61 (18)	5.34 (2. 705–10.52)	

### Antimicrobial susceptibility pattern of isolated pathogens

Isolated pathogens were tested against selected eight antibiotics and the results obtained showed that majority of them were highly sensitive to the antibiotics. Percentage of isolates that were resistant to amoxicillin was 100%, followed by 41% to clarithromycin and 35% to amoxicillin-clavulanic acid. However, all isolates were 100% susceptible to trimethoprim-sulfamethoxazole, ceftriaxone; chloramphenicol, gentamicin, and kanamycin respectively (Table 4).

**Table 4:** Antibiotic susceptibility pattern of pathogens isolated from food handlers at Arba Minch University students’ cafeteria, Arba Minch, South Ethiopia, April–June 2015

Isolates	RXN	Antimicrobial agents (%)							
		GEN	C	SXT	K	AMC	CLR	CRO	AML
*Salmonella Spp* (*n* = 24)	S	24 (100)	24 (100)	24 (100)	24 (100)	16 (66.7)	20 (83.3)	24 (100)	0
	R	0	0	0	0	8 (33.3)	4 (16.7)	0	24 (100)
*Shigella Spp* (*n* = 10)	S	10 (100)	10 (100)	10 (100)	10 (100)	6 (60)	0	10 (100)	0
	R	0	0	0	0	4 (40)	10 (100)	0	10 (100)
Total (*n* = 34)	S	34 (100)	34 (100)	34 (100)	34 (100)	22 (65)	20 (59)	34 (100)	0
	R	0)	0	0	0	12 (35)	14 (41)	0	34 (100)

S = Sensitive R = Resistant, *GEN* Gentamicin, *C* Chloramphenicol, *SXT* Sulphamethoxazole-trimethoprim, *K* Kanamycin, *AMC* Amoxicillin-clavulanic acid, *CLR* Clarithromycin, *CRO* Ceftriaxone, *AML* Amoxicillin

## Discussion

Food handlers may carry a wide range of enteropathogens and participated in the transmission of many infections to the public in the community and to patients in hospitals. The spread of disease via food handlers is a common and persistent problem worldwide [20]. Therefore, this study was undertaken to assess the prevalence, antimicrobial susceptibility patterns and associated risk factors of *Shigella* and *Salmonella* among food handlers in Arba Minch University, South Ethiopia.

In the present study, high prevalence of *Salmonella* and *Shigella* observed in the age group of 21–35 years which was comparable with a study conducted in other region of Ethiopia [8, 21]. Based on the result of current investigation, the prevalence of *Salmonella* infection was higher in females which are supported by the previous studies conducted in Ethiopia [6, 21]. Of the total pathogens isolated, 35.3 and 5.8% were among primary school and illiterate respectively.

About 76.1% of food handlers had a trimmed finger nail which agrees with study of 82.2% in Jimma, Ethiopia [22] but higher than study of 15.2% in Addis Ababa, Ethiopia [21]. Finger nail status had significant association with the isolation rate of pathogens in this study, which may serve as a vehicle for transport of microorganisms from their source to the foods or/and directly in to the body.

It is expected that all food handlers at University, military and hospital cafeterias should have a medical checkup for food-borne pathogens; despite this fact, the interview result of this study showed that none of the food handlers working in Arba Minch University students’ cafeteria had a medical checkup for intestinal infection which agrees with the study done in Bahir Dar town, Ethiopia [8].

In this study, the rate of total pathogens isolation from stool is 10% which is quite lower than 62.6% among food handlers working in Federal Capital Territory of Nigeria [23], 30.1% among suspected asymptomatic food handlers for bacteria and parasite in Omdurman, Sudan [24] and 51% among food handlers in Abeokuta [25], but higher than a study from Japan among food workers in which only 0.032% were positive [26] and 0–13.3% in North India [27]. This rate of detection indicates that, the hygienic condition of the food handlers was challenged by the isolation of enteric pathogens like *Salmonella* from the stool cultures. Good hygiene, both personal and in food handling practices, is the basis for preventing the transmission of pathogens from food handling personnel to consumers (students).

Isolation rate of *Shigella* species among food handlers in this study was 3% which may indicate food handlers’ low hygienic status and may lead to outbreaks of bacillary dysentery among the student population. The prevalence of *Shigella* species in the stools of the food-handlers (3%) was lower with the findings of other studies conducted elsewhere in Nigeria (15.5%) [23]. However, the present result was higher than the reports from Abeokuta 0% [25] and similar with study done in Gondar, Ethiopia 2.7–3.1% [28, 29]. The discrepancy may be due to the difference in technique of pathogens isolation, type of study participant and the sample size.

*Salmonella* isolation rate in this study was 6.9%, however, higher percentage was isolated from study carried out in Nigeria of which *Salmonella* species account 42.3% [23]. The food handlers, from whom *Salmonella* species was isolated, may be a potential risk group for a sudden outbreak of *Salmonella* food poisoning in the students’ population. This finding agrees with a study of 5.5% in Abeokuta [25], and disagree with 3.5% in Addis Ababa University, Ethiopia [21], 3.1% in University of Gondar, Ethiopia [6], 1.6% in Bahir Dar town, North West Ethiopia [8], and 1% in Mekelle, Ethiopia [9] of food handlers were found to be infected. Furthermore, none of the food-handlers were positive for *Salmonella* species in a study done on food-handlers working in the cafeterias of the University of Gondar and the Gondar Teachers Training College, Gondar, Ethiopia [28]. The high *Salmonella* carrier rate of 6.9% may indicate low educational level, poor toilet facilities and lifestyle.

Even though, the present study showed a high rate of isolates sensitivity to majority of antimicrobial agents, high frequency of resistance to amoxicillin, clarithromycin, and amoxicillin-clavulinic acid has been observed. This may be due to availability and indiscriminate (unrestricted) use of the drugs without prescription.

Antimicrobial susceptibility test was done for *Salmonella* species on eight selected antibiotics by disk diffusion technique showed that they tend to be resistant to amoxicillin (100%), amoxicillin-clavulanic acid (33.3%) and clarithromycin (17%). This was consistent with study done in Ethiopia [6].

*Shigella* species was 100% resistant to amoxicillin and clarithromycin whereas, 40% resistant to amoxicillin-clavulunic acid however it indicates high sensitivity to gentamicin, chloramphenicol, Sulphamethoxazole-trimethoprim and kanamycin. This was consistent with the study done in another region of Ethiopia [7].

## Conclusion

In this study, total rate of isolated pathogens from stool is 10% of which *Salmonella* accounts 6.9% and *Shigella* 3%. Of those checked risk factors, finger nail status, hand washing practice after toilet, and transferring food with bare hands were significantly associated with pathogens infection. Therefore, constant epidemiological surveillance, improvement of personal hygiene and environmental sanitation are recommended to control pathogens infection in the food handlers.

## Abbreviations

AOR: Adjusted odd ratio
ATCC: American type culture collection
CDC: Center for disease control and prevention
CI: Confidence interval
CLSI: Clinical laboratory standard institute
SOP: Standard operating procedures
SPSS: Statistical package for social sciences
WHO: World health organization

## References

[1] World Health Organization (2007). Food safety and food borne illness.

[2] World Health Organization (2006). Consultation to Develop a Strategy to Estimate the Global Burden of Foodborne Diseases.

[3] Mukhopadhyay P, Joardar GK, Bag K, Samanta A, Sain S, Koley S (2012). Identifying key risk behaviors regarding personal hygiene and food safety practices of food handlers working in eating establishments located within a hospital campus in Kolkata. Al Ameen J Med Sci.

[4] Zeru K, Kumie A (2007). Sanitary conditions of food establishments in Mekelle town, Tigray, north Ethiopia. Ethiop J Health Dev.

[5] National Food Safety Systems in Africa (2005). A situation analysis.

[6] Garedew-Kifelew L, Wondafrash N, Feleke A (2014). Identification of drug-resistant *Salmonella* from food handlers at the University of Gondar, Ethiopia. BMC Res Notes.

[7] Tiruneh M (2009). Serodiversity and antimicrobial resistance pattern of *Shigella* isolates at Gondar University teaching hospital, Northwest Ethiopia. Jpn J Infect Dis.

[8] Abera B, Biadegelgen F, Bezabih B (2010). Prevalence of Salmonella typhi and intestinal parasites among food handlers in Bahir Dar Town. Northwest Ethiopia. Ethiop J Health Dev..

[9] Nigusse D, Kumie A (2012). Food hygiene practices and prevalence of intestinal parasites among food handlers working in Mekelle university student’s cafeteria, Mekelle. GARJSS.

[10] Birhaneselassie M, Williams D (2013). A study of *Salmonella* carriage among asymptomatic food-handlers in southern Ethiopia. Int J Food Sci Nutr.

[11] Mulet D, Ashenafi M (2001). *Salmonella*, *Shigella* and growth potential of other food- borne pathogens in Ethiopia street vended foods. East Afr Med J.

[12] Girma G (2015). Prevalence, antibiogram and growth potential of *salmonella* and *Shigella* in Ethiopia: implications for public health. Rev Adv Life Sci Tech.

[13] World Health Organization (2004). Global burden of disease (GBD).

[14] Yismaw G, Negeri CAK, Tiruneh M, Mulu A (2007). Antimicrobial resistance pattern of *Salmonella* isolates from Gondar University hosipital, North West Ethiopia. Ethiop Pharm J.

[15] Abera G (2004). Shigellosis in Ethiopia: review of studies conducted since 1974. Ethiop J Biol Sci.

[16] Niyogi SK (2004). Shigellosis. J Microbiol.

[17] Gerbe-yohannes A, Drasar BS (1987). *Shigella* dysentriae and *Shigella* felexineri; sero type prevalence and seasonal distribution in Addis Ababa Ethiopia 1974–85. Ethiop J Health Dev.

[18] Afeworki G, Lirneneh Y (1980). Multiple drug resistance within *Shigella* serogroups. Ethiop Med J.

[19] Roma B, Worku S, T/Mariam S, Langeland N (2000). Antimicrobial susceptibility pattern of *Shigella* isolates in Awassa. Ethiop J of Health Dev.

[20] Mohan V, Dass L, Lal M (2001). An evaluation of health status of food handlers of eating establishments in various educational and health institutions in Amritsar. Indian J comm Med.

[21] Aklilu A, Kahase D, Dessalegn M, Tarekegn N, Gebremichael S, Zenebe S, Desta K, Mulugeta G, Mamuye Y, Mama M (2015). Prevalence of intestinal parasites, *Salmonella* and *Shigella* among apparently health food handlers of Addis Ababa University student’s cafeteria, Addis Ababa, Ethiopia. BMC Res Notes.

[22] Tefera T, Mebrie G. Prevalence and predictors of intestinal parasites among food handlers in Yebu town, southwest Ethiopia. PLoS One. 2013;9(10):e110621–1. 10.1371/journal.pone.0110621 PMC4201565 25329050

[23] Ifeadike C, Ironkwe O, Adogu P, Nnebue C, Emelumadu O, Nwabueze S, Ubajaka C (2012). Prevalence and pattern of bacteria and intestinal parasites among food handlers in the Federal Capital Territory of Nigeria. Niger Med J.

[24] Saeed HA, Hamid HH (2010). Bacteriological and parasitological assessment of food handlers in the Omdurman area of Sudan. J Microbiol Immunol Infect.

[25] Mobolaji OA, Olubunmi OF (2014). Assessment of the hygienic practices and the incidence of enteric bacteria in food handlers in small businesses in an urban area in Abeokuta. Int J Microbiol Res.

[26] Murakami K, Ishihara T, Horikawa K, Oda T (2007). Features of *Salmonella* serovars among food handlers in Kyushu, Japan. Microbiologica-Bologna.

[27] Khurana S, Taneja N, Thapar R, Sharma M, Malla N (2010). Intestinal bacterial and parasitic infections among food handlers in a tertiary care hospital of North India. Trop Gastroenterol.

[28] Andargie G, Kassu A, Moges F, Tiruneh M, Huruy K (2008). Prevalence of bacteria and intestinal parasites among food-handlers in Gondar town, northwest Ethiopia. J Health Popul Nutr.

[29] Dagnew M, Tiruneh M, Moges F, Gizachew M (2013). Bacterial profile and antimicrobial susceptibility pattern among food handlers at Gondar University Cafeteria, Northwest Ethiopia. J Infect Dis Ther.

